# Species-Specific Deamidation of RIG-I Reveals Collaborative Action between Viral and Cellular Deamidases in HSV-1 Lytic Replication

**DOI:** 10.1128/mBio.00115-21

**Published:** 2021-03-30

**Authors:** Huichao Huang, Jun Zhao, Ting-Yu Wang, Shu Zhang, Yuzheng Zhou, Youliang Rao, Chao Qin, Yongzhen Liu, Yongheng Chen, Zanxian Xia, Pinghui Feng

**Affiliations:** a Section of Infection and Immunity, Herman Ostrow School of Dentistry, Norris Comprehensive Cancer Center, University of Southern California, Los Angeles, California, USA; b Department of Oncology, NHC Key Laboratory of Cancer Proteomics, XiangYa Hospital, Central South University, Changsha, Hunan, China; c Department of Cell Biology, Hunan Key Laboratory of Animal Models for Human Diseases, Hunan Key Laboratory of Medical Genetics and Center for Medical Genetics, School of Life Sciences, Central South University, Changsha, Hunan, China; UC Berkeley

**Keywords:** HSV-1 UL37, innate immune defense, RIG-I, deamidation, glutamine amidotransferase, herpesvirus, immune evasion, phosphoribosyl pyrophosphate amidotransferase

## Abstract

Retinoic acid-inducible gene I (RIG-I) is a sensor that recognizes cytosolic double-stranded RNA derived from microbes to induce host immune response. Viruses, such as herpesviruses, deploy diverse mechanisms to derail RIG-I-dependent innate immune defense. In this study, we discovered that mouse RIG-I is intrinsically resistant to deamidation and evasion by herpes simplex virus 1 (HSV-1). Comparative studies involving human and mouse RIG-I indicate that N495 of human RIG-I dictates species-specific deamidation by HSV-1 UL37. Remarkably, deamidation of the other site, N549, hinges on that of N495, and it is catalyzed by cellular phosphoribosylpyrophosphate amidotransferase (PPAT). Specifically, deamidation of N495 enables RIG-I to interact with PPAT, leading to subsequent deamidation of N549. Collaboration between UL37 and PPAT is required for HSV-1 to evade RIG-I-mediated antiviral immune response. This work identifies an immune regulatory role of PPAT in innate host defense and establishes a sequential deamidation event catalyzed by distinct deamidases in immune evasion.

## Abstract (precis)

Herpesviruses are ubiquitous pathogens in human and establish lifelong persistence despite host immunity. The ability to evade host immune response is pivotal for viral persistence and pathogenesis.

## INTRODUCTION

Innate immunity is the first line of defense that limits the propagation of invading pathogens. Pattern recognition receptors sense microbe-associated molecular patterns that are often structural components or replication intermediates of microbes (1). Retinoic acid-induced gene I (RIG-I) is a cytosolic RNA sensor that recognizes microbial double-stranded RNA carrying 5′ tri- or diphosphate moiety (2–4). Structural studies have provided snapshots of distinct conformations that purport an elegant model of RIG-I activation induced by RNA (5, 6). Activated RIG-I undergoes homodimerization and heterodimerization with the mitochondrial antiviral signaling (MAVS; also known as IPS, VISA, and CARDIF) (7–10) adaptor via an exposed caspase activation and recruitment domains (CARDs) (11), triggering MAVS oligomerization (12) and downstream signaling cascades that culminate in antiviral cytokine production to establish a potent antiviral state.

Viruses are obligate intracellular parasites that rely primarily on cellular machinery for their replication. Large DNA or RNA viruses, such as herpesviruses and coronaviruses, produce a number of polypeptides that are nonessential for their replication. These viral gene products often modulate the cellular environment in preparation for viral replication. Innate immune response is one key constituent of host defense that viruses evolve to evade, deflect, and explore for their own benefit. Viruses have evolved diverse means to counteract host innate immune response (13, 14). Although RIG-I is appreciated for its roles in host defense against RNA viruses (15), it is also pivotal in restricting herpesviruses that carry DNA genomes (16–21) in macrophages (22) and other types of cells. Conversely, herpesviruses display distinct mechanisms to suppress RIG-I-mediated innate immune signaling (16, 23–26). One mechanism that we previously reported is achieved via protein deamidation of key signaling molecules, such as RIG-I and cyclic GMP-AMP synthase (cGAS) (16, 27).

Protein deamidation is the hydrolysis of the side chain of asparagine or glutamine residues of proteins. The prevailing notion is that protein deamidation, particularly of asparagines, is a nonenzymatic reaction associated with protein functional decay or “aging” (28). As such, functions of protein deamidation are poorly characterized. In mammalian cells, protein deamidation can be catalyzed by a few recently defined enzymes, which include bacterial secreted effectors and cellular glutamine amidotransferases (GATs) (29). Bacterial secreted effectors can function as *bona fide* deamidases, sharing structures similar to cysteine proteases (30–32). In contrast, GATs are originally known to catalyze the synthesis of nucleotides, amino acids, glycoproteins, and an enzyme cofactor (NAD), which are building blocks of proliferating cells and replicating viruses (33). The activity of cellular GATs to deamidate key signaling proteins (e.g., RIG-I, cGAS, and RelA) in innate immune response (27, 35, 41) suggests that signaling pathways are coupled to the metabolic status of a cell, and that viruses may exploit such a mechanism to evade host defense.

We have previously reported that herpesviruses deploy protein deamidation to evade innate immune defense via targeting specific cytosolic sensors, such as RIG-I and cGAS (16, 27, 41). While gammaherpesviruses encode viral pseudoenzymes (known as vGATs) to deamidate RIG-I (41), herpes simplex virus 1 UL37 serves as a genuine enzyme to deamidate and inactivate RIG-I and cGAS (16, 27). Interestingly, both RIG-I and cGAS are deamidated at multiple sites, and the molecular details of these deamidation events are unknown. Examining the species-specific deamidation of RIG-I by HSV-1 UL37, we discovered that HSV-1 UL37 deamidates N495 of human RIG-I (hRIG-I), but not the equivalent of mouse RIG-I (mRIG-I). Further analysis determined that cellular phosphoribosylpyrophosphate amidotransferase (PPAT), the rate-limiting enzyme of the *de novo* purine synthesis pathway (36), targets N549 for deamidation. Thus, collaborative action of viral UL37 and cellular PPAT enables the deamidation and evasion of RIG-I in HSV-1 infection. This study not only identifies a new deamidase in innate immune regulation, but also uncovers molecular coordination between two distinct enzymes in protein deamidation and viral immune evasion.

## RESULTS

### Mouse RIG-I more potently restricts HSV-1 lytic replication than human RIG-I.

**FIG 1:**
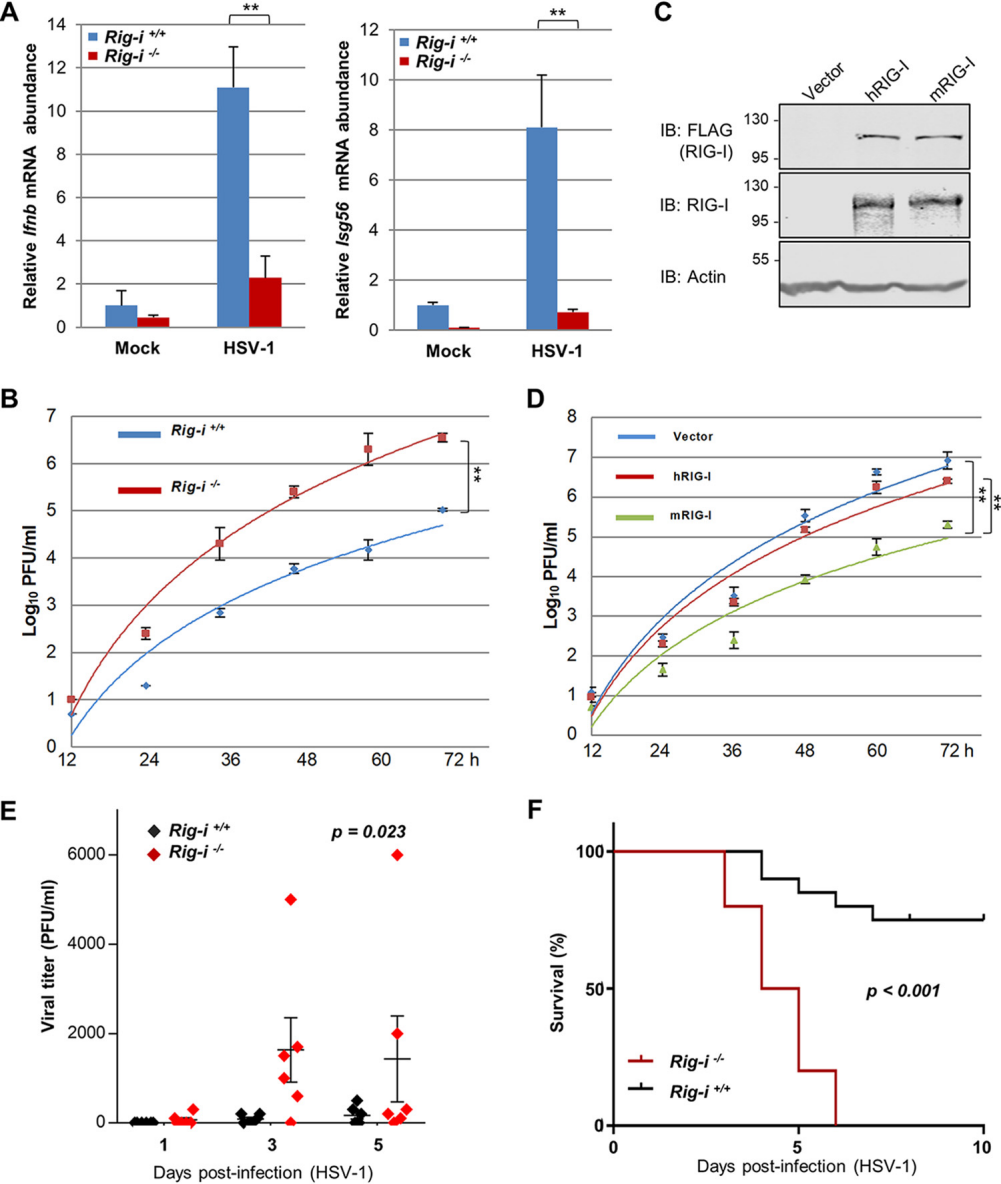
Antiviral activity of mouse and human RIG-I in HSV-1 infection. (A and B) RIG-I wild-type or knockout mouse embryonic fibroblasts (MEFs) were infected with HSV-1 (MOI = 2) and total RNA was extracted to analyze *Ifnb* and *Isg56* gene expression (A). Viral titer was determined by plaque assay (B). (C and D) The expression of human and mouse RIG-I (hRIG and mRIG) was reconstituted via lentivirus infection in *Rig-i^−/−^* MEFs analyzed by immunoblotting (IB) (C), and HSV-1 replication (MOI = 0.01) was determined as in panel D. (E and F) Age- and gender-matched RIG-I wild-type and knockout mice were infected with HSV-1 (1,000 PFU) via the ocular route. Viral load in eye swabs was determined by plaque assay (E). Alternatively, mice were infected with lethal dose of HSV-1 (5 × 10^7^ PFU) intravenously, and mouse survival was recorded over time (F). Data are presented as mean ± standard deviation (SD). Significance was calculated using an unpaired (paired for Fig. 1B and D) two-tailed Student’s *t* test. **, *P < *0.01; ***, *P < *0.001; NS, nonsignificant. Two-way analysis of variance (ANOVA) test and log-rank (Mantel-Cox) test were used to calculate the *P* values for Fig. 1E and F, respectively, using GraphPad Prism.

We have recently reported that HSV-1 deploys UL37 to deamidate and inactivate RIG-I. Specifically, UL37 induces the deamidation of N495 and N549 in the helicase domain, and deamidation of these two residues results in the loss of RNA binding. To determine the role of deamidation in RIG-I-mediated innate immune response *in vivo*, we probed the antiviral activity of RIG-I using mouse embryonic fibroblasts (MEFs) and mice deficient in RIG-I. We first infected RIG-I wild-type and knockout MEFs with a high dose of HSV-1 (multiplicity of infection [MOI] = 5) and examined the expression of beta interferon (IFN-β) and ISG56. Strikingly, loss of RIG-I greatly reduced the expression of these two antiviral genes in MEFs (Fig. 1A). Similar results were observed in MEFs deficient in MAVS, supporting the antiviral activity of the RIG-I-MAVS pathway against HSV-1 infection (see Fig. S1A in the supplemental material). Conversely, loss of RIG-I increased the expression of lytic genes of HSV-1 (Fig. S1B). Our previous study has shown that loss of RIG-I had no apparent effect on antiviral gene expression in human foreskin fibroblasts (HFFs) when a high MOI of HSV-1 was used, due to the deamidation and consequent evasion of RIG-I (16). We further examined the replication kinetics of HSV-1 in RIG-I wild-type and knockout MEFs and found that loss of RIG-I significantly increased HSV-1 replication in MEFs (Fig. 1B). To compare the antiviral activity of human RIG-I (hRIG-I) and mouse RIG-I (mRIG-I), we “reconstituted” RIG-I expression in RIG-I-deficient MEFs via lentiviral infection. Immunoblotting analysis indicates that hRIG-I and mRIG-I were expressed at similar levels in these “reconstituted” cells (Fig. 1C). When HSV-1 lytic replication was examined, we found that mRIG-I reduced HSV-1 titer by approximately 1 order of magnitude (Fig. 1D). The expression of hRIG-I had a marginal effect on HSV-1 replication. This result shows that mRIG-I restricts HSV-1 lytic replication much more potently than hRIG-I.

10.1128/mBio.00115-21.1 FIG S1 Related to Fig. 1. (A) *Ifnb* and *Isg56* abundance in mitochondrial antiviral signaling (MAVS) wild-type or knockout mouse embryonic fibroblasts (MEFs) infected with herpes simplex virus 1 (HSV-1) (multiplicity of infection [MOI] = 2) for 16 h. (B) HSV-1 *UL29* and *UL54* abundance in RIG-I wild-type or knockout MEFs infected with HSV-1 (MOI = 2) for 16 h. Download FIG S1, PDF file, 0.2 MB. Copyright © 2021 Huang et al. 2021 Huang et al. https://creativecommons.org/licenses/by/4.0/ This content is distributed under the terms of the Creative Commons Attribution 4.0 International license.

To probe the *in vivo* role of RIG-I in HSV-1 infection, we infected wild-type and RIG-I-knockout mice via ocular scratching and evaluated viral shedding in the eye. As shown in Fig. 1E, HSV-1 had significantly higher titer in the eye of RIG-I-knockout mice than in that of RIG-I wild-type mice. Furthermore, when mice were infected with a lethal dose of HSV-1, all RIG-I-knockout mice succumbed to HSV-1 infection by day 6 postinfection, while 75% of RIG-I wild-type mice survived (Fig. 1F). These results demonstrate that RIG-I is an antiviral molecule and restricts HSV-1 replication in mice.

### Mouse RIG-I is resistant to UL37-mediated deamidation.

**FIG 2:**
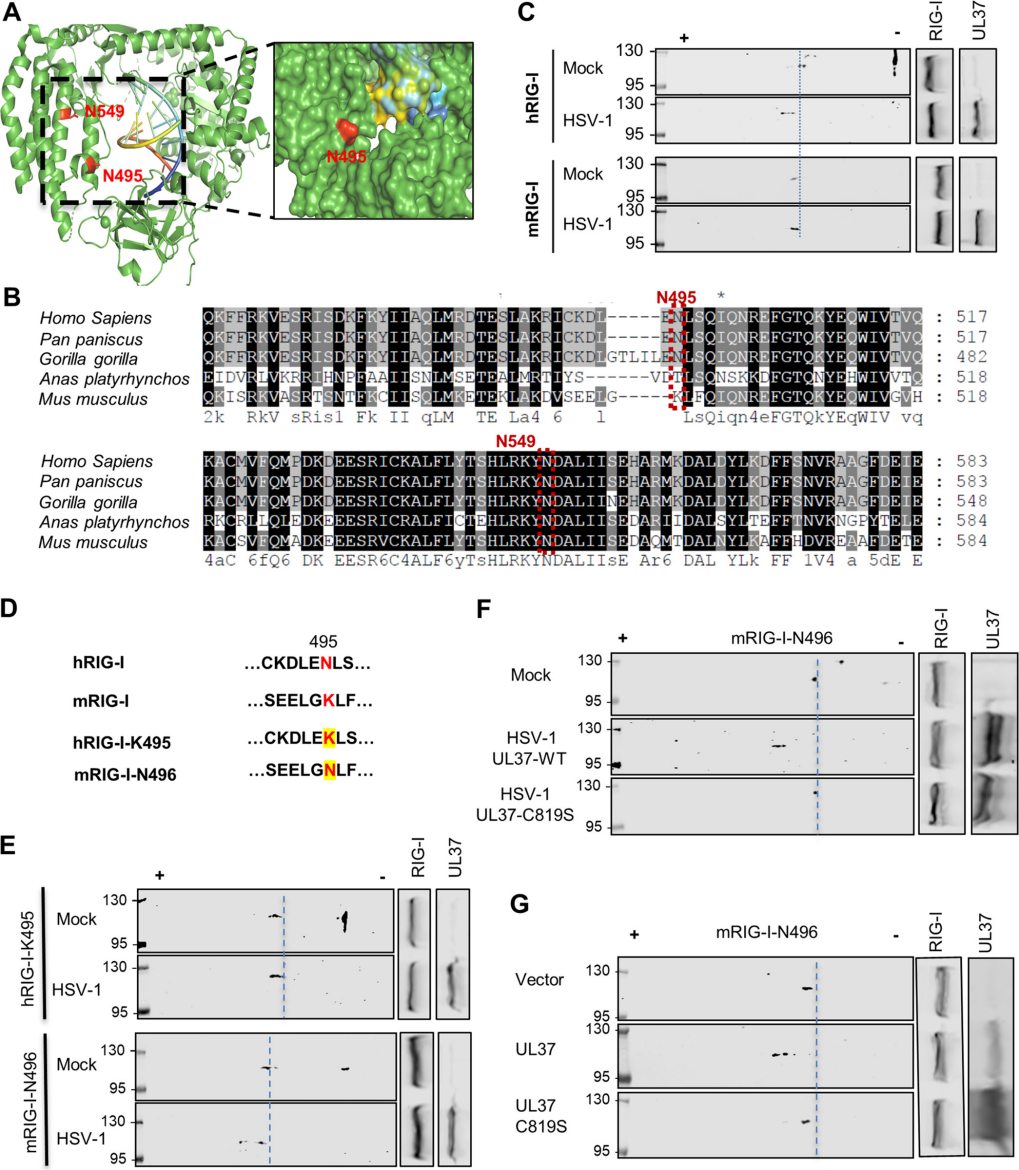
Comparative analysis indicates that N495 of human RIG-I dictates the deamidation by HSV-1 UL37. (A) Structure and surface of the helicase domain of hRIG-I with N495 and N549 highlighted (PDB identifier 3TMI). (B) Sequences flanking N495 and N549 of RIG-I across multiple species were aligned, with N495 and N549 (of human RIG-I) highlighted in red boxes. (C) 293T cells stably expressing hRIG-I (top) or mRIG-I were infected with HSV-1 (MOI = 2) and whole-cell lysates were analyzed by two-dimensional gel electrophoresis or regular SDS-PAGE, followed by immunoblotting with indicated antibodies. (D) Alignment of hRIG-I and mRIG-I sequences flanking N495 of hRIG-I and K496 of mRIG-I (top). Highlighted residues were mutated to create the hRIG-I containing N495K mutation (hRIG-I-K495) and the mRIG-I containing K496N mutation (mRIG-I-N496) (bottom). (E) 293T cells stably expressing hRIG-I-K495 or mRIG-I-N496 were infected with HSV-1 (multiplicity of infection [MOI] = 2). At 16 h postinfection, whole-cell lysates were prepared and analyzed by two-dimensional gel electrophoresis, followed by immunoblotting with indicated antibodies. (F and G) 293T cells stably expressing mRIG-I-N496 were infected with HSV-1 carrying UL37 or UL37C819S mutant (MOI = 2) (F) or were transfected with empty vector or vector containing UL37 or UL37C819S mutant (G). At 16 h postinfection or 30 h posttransfection, whole-cell lysates were analyzed as above to assess the charge status of mRIG-I-N496 with indicated antibodies.

We have previously reported that HSV-1 UL37 specifically induces the deamidation of N495 and N549 in the helicase domain of hRIG-I. The three-dimensional structure of the helicase domain indicates that N495 is exposed on the surface, while N549 is more buried within the helicase domain (Fig. 2A), raising the question of how these two deamidation sites are accessed by UL37. Sequence alignment indicates that human and nonhuman primate RIG-I proteins contain N495, while RIG-I proteins of mice and other mammals contain residues other than N or Q, including K in mRIG-I (Fig. 2B). mRIG-I restricts HSV-1 infection much more potently than hRIG-I, suggesting that mRIG-I is resistant to evasion by HSV-1. These observations suggest that N495 is likely important for RIG-I deamidation by UL37. To test this hypothesis, we examined the deamidation of hRIG-I and mRIG-I by two-dimensional gel electrophoresis using 293T cells stably expressing hRIG-I and mRIG-I. Consistent with what we reported before, HSV-1 infection induced an apparent shift of hRIG-I toward the positive pole of the gel strip, indicative of deamidation (Fig. 2C). In stark contrast, mRIG-I was not shifted by HSV-1 infection under the same conditions (Fig. 2C). These results support the conclusion that mRIG-I is resistant to HSV-1-induced deamidation.

### N495 of human RIG-I confers resistance to UL37-mediated deamidation.

To determine the importance of N495 in RIG-I deamidation, we asked whether N495 is required for hRIG-I deamidation induced by HSV-1. We created the hRIG-I mutant containing an N495K mutation, referred to as hRIG-I-K495 (Fig. 2D). Conversely, we created an mRIG-I mutant containing K496N, referred to as mRIG-I-N496. 293T cells stably expressing hRIG-I-K495 were infected with HSV-1, and RIG-I was examined by two-dimensional gel electrophoresis. We found that HSV-1 infection failed to shift hRIG-I-K495 when UL37 was robustly expressed. This result showed that hRIG-I-K495 is resistant to HSV-1-induced deamidation (Fig. 2E). Under similar conditions, a fraction of mRIG-I-N496 was shifted toward the positive pole of the gel strip, although a significant portion was not shifted, indicating that the HSV-1-induced deamidation was incomplete (Fig. 2E). Thus, these results indicate that N495 of hRIG-I and the corresponding K496 site of mRIG-I dictate the deamidation of RIG-I induced by HSV-1.

To assess the role of UL37 in the deamidation of mRIG-I-N496, we analyzed mRIG-I-N496 deamidation with the UL37 wild type and the deamidase-deficient UL37C819S mutant. mRIG-I-N496 was examined under conditions of HSV-1 infection and UL37 expression. Two-dimensional gel electrophoresis showed that the infection with recombinant HSV-1 UL37 wild type, but not that with the deamidase-deficient HSV-1 UL37C819S mutant, shifted mRIG-I-N496 to the positive pole of the gel strip (Fig. 2F). Similarly, the expression of wild-type UL37, but not that of the UL37C819S mutant, shifted mRIG-I-N496 toward the positive side of the gel strip (Fig. 2G). Consistent with these deamidation results, mRIG-I, but not mRIG-I-N496, increased antiviral cytokine expression in response to HSV-1 infection and conversely reduced the expression of viral lytic genes (see Fig. S2A and B in the supplemental material). Taken together, these results collectively show that UL37 is responsible for inducing mRIG-I-N496 deamidation under conditions of ectopic expression and during HSV-1 infection.

**FIG 3:**
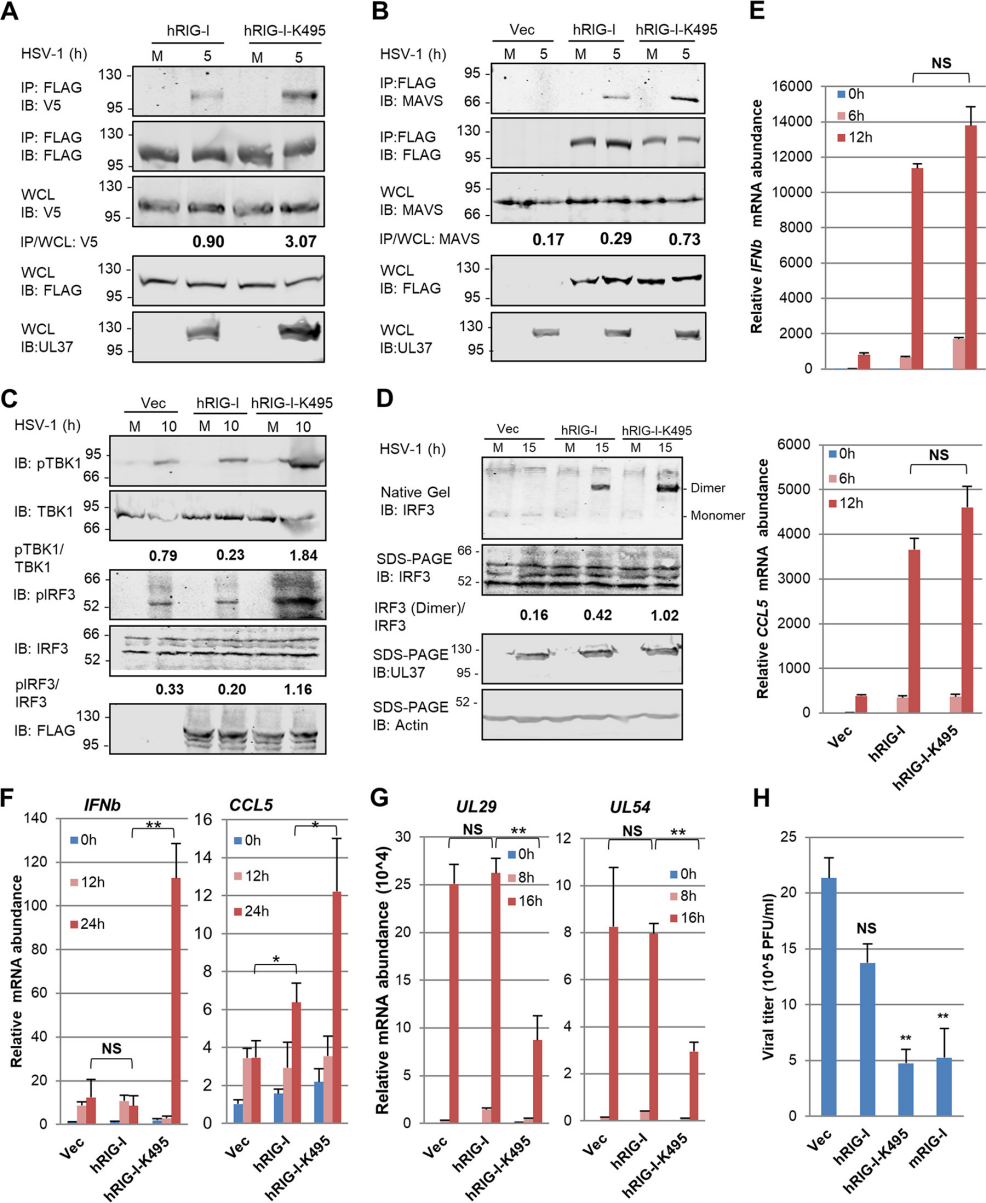
hRIG-I-K495 is more potent than the hRIG-I wild-type in host defense in response to HSV-1 infection. (A) 293T cells stably expressing FLAG-RIG-I (293T/FLAG-RIG-I) were transfected with plasmids containing the V5-tagged hRIG-I wild type or RIG-I-K495. At 24 h posttransfection, cells were infected with HSV-1 (MOI = 2) for 5 h. Whole-cell lysates were precipitated with anti-FLAG (RIG-I). Precipitated proteins and WCLs were analyzed by immunoblotting with indicated antibodies. (B) 293T/FLAG-RIG-I cells were infected with HSV-1 (MOI = 2) for 5 h. WCLs were precipitated with anti-FLAG (RIG-I), precipitated proteins and whole-cell lysates (WCLs) were analyzed by immunoblotting with indicated antibodies. (C and D) 293T/FLAG-RIG-I cells were infected with HSV-1 (MOI = 2) for 10 (C) and 15 h (D). WCLs were prepared and analyzed by immunoblotting with indicated antibodies for phosphorylation of TBK-1 and IRF3 (C) and for IRF3 dimerization by native gel electrophoresis (D). (E and F) Control 293T cells (vector) or those stably expressing the hRIG-I wild type (WT) or hRIG-I-K495 were infected with Sendai virus (100 hemagglutinating units [HAU]/ml) (E) or HSV-1 (MOI = 2) (F) for the indicated time. Total RNA was extracted and analyzed by reverse transcription and real-time PCR with primers specific for *Ifnb* and *Ccl5*. (G and H) 293T cells as described in panel E were infected with HSV-1 (MOI = 0.1). Total RNA was extracted and reverse transcribed, and quantified by real-time PCR with primers specific for HSV-1 UL29 and UL54 genes (G). Viral titer was determined by plaque assay on Vero monolayer (H). Data are presented as mean ± SD. Significance was calculated using an unpaired, two-tailed Student’s *t* test. **, *P < *0.01; ***, *P < *0.001; NS, nonsignificant.

10.1128/mBio.00115-21.2 FIG S2 Related to Fig. 3. (A) *IFN-β* and *CXCL10* abundance in the 293T cells stably expressing the mouse RIG-I wild type (MWT) or mRIG-I-N496 (MKN) infected with HSV-1 (MOI = 2) for 16 h. (B) HSV-1 *UL29* and *UL54* abundance in cells as described in panel A and infected with HSV-1 (MOI = 2) for 16 h. Download FIG S2, PDF file, 0.2 MB. Copyright © 2021 Huang et al. 2021 Huang et al. https://creativecommons.org/licenses/by/4.0/ This content is distributed under the terms of the Creative Commons Attribution 4.0 International license.

### hRIG-I-K495 more robustly activates innate immune response upon HSV-1 infection.

To assess the biological significance of the deamidation-resistant hRIG-I-K495 mutant, we examined the activation of the RIG-I-MAVS-IFN pathway. These signaling events include the homodimerization and heterodimerization with MAVS of RIG-I, phosphorylation of TBK-1, phosphorylation and dimerization of IRF3, and cytokine gene expression. We used 293T cells stably expressing the hRIG-I wild type and the hRIG-I-K495 mutant for this experiment, because endogenous hRIG-I can be deamidated and inactivated by HSV-1 UL37 when cells are infected with a high MOI (e.g., MOI = 5). To evaluate RIG-I dimerization, we established 293T cells stably expressing FLAG-RIG-I and RIG-I-V5. Upon HSV-1 infection, we found that hRIG-I-K495 demonstrated a higher level of dimerization than that of the hRIG-I wild type (Fig. 3A). Consistent with this result, hRIG-I-K495 demonstrated greater interaction with MAVS than did the hRIG-I wild type, as analyzed by coimmunoprecipitation assay (Fig. 3B). Furthermore, whereas exogenously expressed hRIG-I wild type only marginally increased or had no effect on the phosphorylation of TBK-1 and IRF3, expression of hRIG-I-K495 clearly increased the phosphorylation of TBK-1 and IRF3 (Fig. 3C). The increase in phosphorylated IRF3 also correlated with elevated levels of dimerized IRF3, as analyzed by native gel electrophoresis (Fig. 3D). When antiviral cytokine gene expression in response to Sendai virus infection was examined, hRIG-I-K495 demonstrated a slightly higher, but not statistically significant, activity to induce *IFNB* and *CCL5* expression (Fig. 3E). In contrast, hRIG-I-K495 induced much higher levels of *IFNB* gene expression than did the hRIG-I wild type in response to HSV-1 infection (Fig. 3F). Higher levels of *CCL5* gene expression were also detected in cells with hRIG-I-K495 than in those with hRIG-I. Conversely, the expression of hRIG-I-K495, but not that of hRIG-I, reduced HSV-1 lytic gene expression (Fig. 3G) and infectious virus production (Fig. 3H). Finally, the hRIG-I-K495 mutant inhibited HSV-1 lytic replication as potently as mRIG-I. Taken together, the deamidation-resistant hRIG-I-K495 induces a more robust antiviral immune response than that of the hRIG-I wild type to restrict HSV-1 lytic replication.

### RIG-I-D495 is deamidated at N549 in cells.

**FIG 4:**
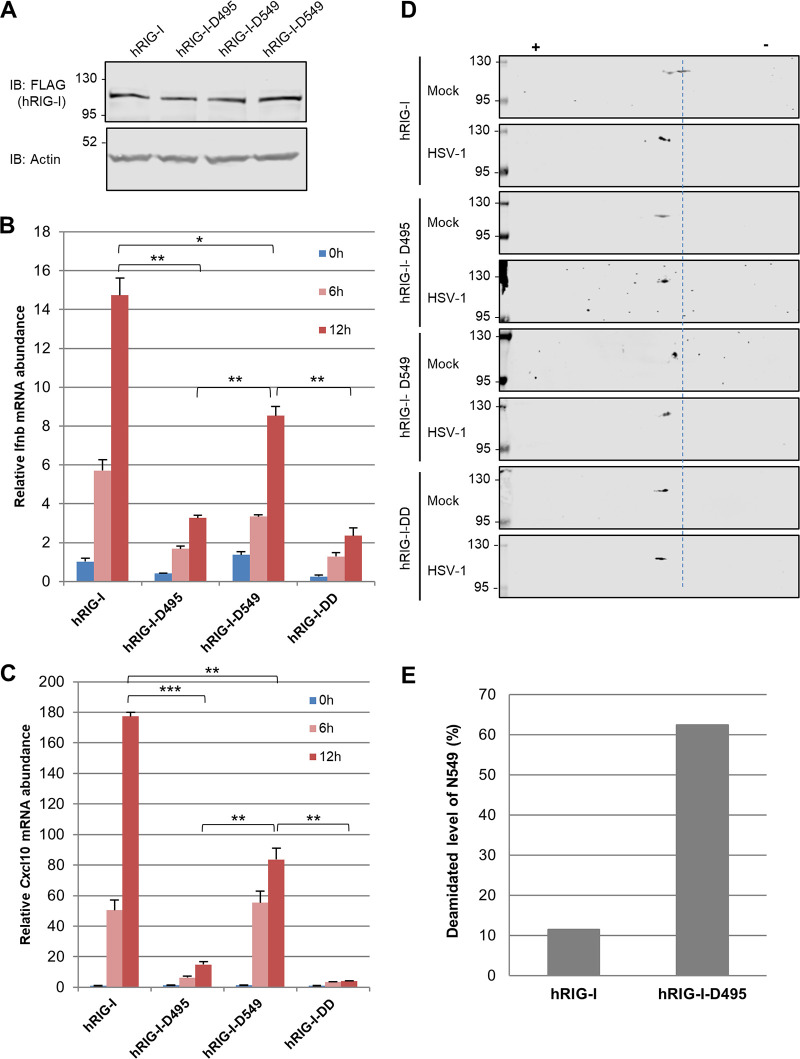
RIG-I-D495 is deamidated at N549 in cells. (A). RIG-I-deficient mouse embryonic fibroblasts (MEFs) were “reconstituted” with lentivirus containing the hRIG-I wild type (WT), hRIG-I-D495, hRIG-I-D549, and hRIG-I-DD. Whole-cell lysates (WCLs) were analyzed with immunoblotting with anti-FLAG (RIG-I) and β-actin. (B and C) *Ifnb* (C) and *Cxcl10* (D) abundance in “reconstituted” *Rig-i^−/−^* MEFs as described in panel A and infected with Sendai virus (100 hemagglutinating units [HAU]/ml) for the indicated time. (D) 293T cells stably expressing the human RIG-I wild type, hRIG-I-D495, hRIG-I-D549, or hRIG-I-DD were infected with HSV-1 (MOI = 2) for 16 h, and WCLs were analyzed by two-dimensional gel electrophoresis and immunoblotting. (E) Percentage of the N549 deamidated peptides of the human RIG-I wild type or hRIG-I-D495 that were purified from stable 239T cells and analyzed by tandem mass spectrometry. Data are presented as mean ± SD. Significance was calculated using an unpaired two-tailed Student’s *t* test. *, *P < *0.05; **, *P < *0.01; ***, *P < *0.001; NS, nonsignificant.

Previously, we found that both N495 and N549 were deamidated during HSV-1 infection, while the single-deamidation mutant N549D (hRIG-I-D549), but not hRIG-I-D495, severely impaired its double-stranded RNA (dsRNA)-binding ability by an *in vitro* RNA gel shift assay, when RIG-I proteins purified from transiently transfected 293T cells were assessed (16). This result is consistent with N549 being highly conserved in all RIG-I proteins across diverse species. Moreover, a close inspection of the RNA-bound structure of RIG-I suggests more direct impact imposed by the deamidation of N549 on RNA binding of RIG-I. Thus, we examined the impact of individual deamidation on RIG-I-mediated innate immune activation. We “reconstituted” RIG-I-deficient MEF with lentivirus containing the hRIG-I wild type, hRIG-I-D495, hRIG-I-D549, or hRIG-I-DD. Immunoblotting analysis indicated that these RIG-I proteins were expressed at similar levels (Fig. 4A). We then analyzed RIG-I activation in these reconstituted RIG-I-deficient MEF cell lines by quantifying the expression of *Ifnb* and *Cxcl10* in response to Sendai virus infection. Reconstituted expression of RIG-I induced robust expression of *Ifnb* and *Cxcl10*, while hRIG-I-D549 had a reduced expression of both genes (Fig. 4B and C). These results are consistent with the suspected role of N549 in RNA binding of RIG-I. Surprisingly, cells expressing hRIG-I-D495 had minimal expression of *Ifnb* and *Cxcl10* in response to Sendai virus infection, demonstrating phenotypes similar to those expressing hRIG-I-DD. This result suggests that hRIG-I-D495 is likely deamidated at N549. Collectively, these results show that hRIG-I-D495, similarly to hRIG-I-DD, is highly impaired in provoking an innate immune response.

To determine whether hRIG-I-D495 is deamidated at N549 in the absence of HSV-1 infection, we examined hRIG-I-D495 and RIG-I-D549 by two-dimensional gel electrophoresis in comparison to the hRIG-I wild type without and with HSV-1 infection. The RIG-I-DD mutant served as a positive control for deamidation. While HSV-1 shifted the hRIG-I wild type toward the positive pole of the gel strip, hRIG-I-D495 migrated to the same location as hRIG-I-DD under the condition of mock infection. Moreover, HSV-1 infection did not further shift hRIG-I-D495, nor RIG-I-DD (Fig. 4D). These results suggest that RIG-I-D495 is deamidated at N549 in the absence of HSV-1 infection. In contrast, hRIG-I-D549 migrated to a position between the hRIG-I wild type and hRIG-I-D495, and it was further shifted by HSV-1 infection to the exact location of hRIG-I-D495. Finally, hRIG-I-DD migrated to the exact location of hRIG-I-D495 and was not further shifted by HSV-1 infection. These results collectively suggest that hRIG-I-D495 is deamidated at N549, even without HSV-1 infection, and that HSV-1 induces the deamidation of N495 of hRIG-I.

To validate the deamidation of N549 in the hRIG-I-D495 mutant, we purified the hRIG-I wild type and hRIG-I-D495 from stable 239T cell lines and analyzed them by tandem mass spectrometry (see Fig. S3A in the supplemental material). We found that ∼10% of the hRIG-I wild type was deamidated at N549, whereas approximately 60% of hRIG-I-D495 was deamidated at N549 under similar conditions (Fig. 4E). In contrast, when hRIG-I and hRIG-I-D549 were purified and analyzed by mass spectrometry, we found that deamidation of N495 was at basal levels, and that of hRIG-I-D549 was slightly lower than that of the hRIG-I wild type (Fig. S3B and C). These results further support the conclusion that deamidation of N495 promotes that of N549, but not *vice versa*.

10.1128/mBio.00115-21.3 FIG S3 Related to Fig. 4. (A and B) Coomassie blue staining of the human RIG-I wild type and indicated mutants purified from 239T stable cells and analyzed by SDS-PAGE. (C) Percentage of the N495 deamidated peptides of the human RIG-I wild type or D549 purified from stable 239T cells and analyzed by tandem mass spectrometry. Download FIG S3, PDF file, 0.3 MB. Copyright © 2021 Huang et al. 2021 Huang et al. https://creativecommons.org/licenses/by/4.0/ This content is distributed under the terms of the Creative Commons Attribution 4.0 International license.

### PPAT targets N549 of hRIG-I for deamidation.

**FIG 5:**
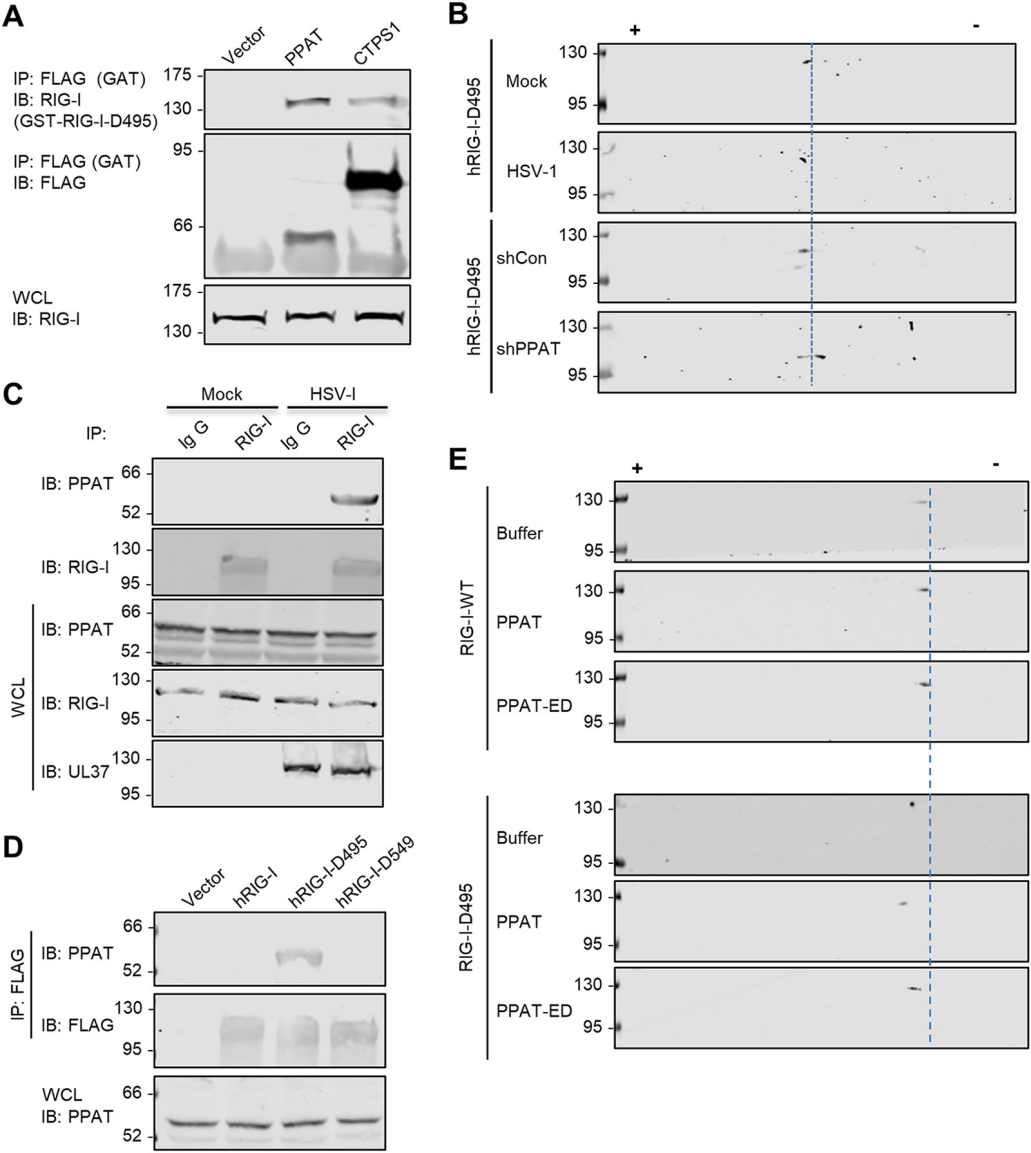
PPAT targets N549 of human RIG-I for deamidation. (A) 293T cells were transiently transfected with plasmids containing glutathione *S*-transferase (GST)-tagged RIG-I-D495 and FLAG-tagged GATs (CTPS1 and PPAT). Whole-cell lysates (WCLs) were precipitated with anti-FLAG (GAT). Precipitated proteins and WCLs were analyzed by immunoblotting with indicated antibodies. (B) 293T cells stably expressing hRIG-I-D495 were infected with lentivirus containing short hairpin RNA (shRNA) control (shCon) or PPAT shRNA (shPPAT) for 48 h, then infected with HSV-1 (MOI = 2) for 12 h. WCLs were analyzed by two-dimensional gel electrophoresis and immunoblotting. (C) 293T cells were mock infected or infected with HSV-1 (MOI = 1) for 12 h. WCLs were precipitated with anti-RIG-I or control antibody, precipitated proteins, and WCLs were analyzed by immunoblotting with indicated antibodies. (D) WCLs of 293T cells stably expressing the hRIG-I wild type, hRIG-I-D495, or hRIG-I-D549 were precipitated with anti-FLAG (RIG-I). WCLs and precipitated proteins were analyzed by immunoblotting with the indicated antibodies. (E) *In vitro* deamidation reaction purified substrate (GST-hRIG-I or GST-hRIG-I-D495), with deamidase (PPAT or PPAT-ED) were analyzed by two-dimensional gel electrophoresis and immunoblotting with anti-GST (RIG-I) antibody.

The observation that hRIG-I-D495 is highly deamidated at N549 in the absence of HSV-1 infection suggests the existence of cellular deamidase(s). To identify the cellular deamidase that targets N549 of hRIG-I-D495 for deamidation, we screened cellular glutamine amidotransferases that interact with hRIG-I-D495 by coimmunoprecipitation assay. The human genome encodes 11 glutamine amidotransferases that catalyze the synthesis of nucleotides, amino acids, glycoproteins, and an enzyme cofactor (NAD). These GAT domain-containing enzymes are potential deamidases in cells, and we previously reported that PFAS and CAD demonstrate intrinsic deamidase activity toward proteins. With the cDNA expression plasmids for all cellular GATs, we found by coimmunoprecipitation assay that hRIG-I-D495 interacted with PPAT, CTPS1, and CAD in transfected 293T cells (Fig. 5A; see also Fig. S4A in the supplemental material). Importantly, the closely related CTPS2 did not interact with hRIG-I-D495, while CTPS1 did, supporting the specificity of these interactions. To determine whether PPAT, CTPS1, or CAD functions as a deamidase of hRIG-I-D495, we depleted these three GATs with short hairpin RNA (shRNA) (Fig. S4B) and examined hRIG-I by two-dimensional gel electrophoresis under HSV-1 infection conditions. This analysis showed that knockdown of PPAT shifted hRIG-I-D495 toward the negative pole of the gel strip, suggesting that PPAT is the deamidase targeting N549 of hRIG-I (Fig. 5B). In contrast, depletion of CTPS1 resulted in a minor shift of hRIG-I-D495 toward the positive side of the gel strip, which is opposite to what we expected, while depletion of CAD had a minor effect on hRIG-I-D495 (Fig. S4C). Furthermore, coimmunoprecipitation assay using endogenous proteins demonstrated that hRIG-I interacted with PPAT only in HSV-1-infected cells, but not in mock-infected cells (Fig. 5C), suggesting that HSV-1 infection induces RIG-I interaction with PPAT.

10.1128/mBio.00115-21.4 FIG S4 Related to Fig. 5. (A) Immunoblots of glutamine amidotransferases (GATs) from the anti-FLAG (top panels) or anti-V5 (bottom) precipitated whole-cell lysates (WCLs) of 293T cells stably expressing FLAG- or V5-tagged RIG-I transfected with indicated GATs. (B) Immunoblots of indicated glutamine amidotransferases (GATs) from whole-cell lysates (WCLs) of 293T/RIG-I stable cells transfected with control or shRNA targeting the GATs for 72 h. (C) Immunoblots of RIG-I-D495 from the whole-cell lysates (WCLs) of 293T stable cells depleted for CTPS1 or CAD and then infected with HSV-1 (MOI = 2) for 16 h, as analyzed by two-dimensional gel electrophoresis. (D) 293T cells were transiently transfected with plasmids containing FLAG-hRIG-I-D495 and FLAG-hRIG-I-DD. Whole-cell lysates (WCLs) were analyzed by two-dimensional gel electrophoresis. Download FIG S4, PDF file, 0.3 MB. Copyright © 2021 Huang et al. 2021 Huang et al. https://creativecommons.org/licenses/by/4.0/ This content is distributed under the terms of the Creative Commons Attribution 4.0 International license.

To test the hypothesis that HSV-1 infection and UL37 deamidation induces hRIG-I interaction with PPAT, we first probe the interaction of PPAT with the RIG-I wild type and deamidated mutants, including hRIG-I-D495 and hRIG-I-D549. As shown in Fig. 5D, we found that hRIG-I-D495, but not the RIG-I wild type and RIG-I-D549, interacted with PPAT. This result suggests that deamidation of N495 induces hRIG-I to interact with PPAT, leading to the subsequent deamidation of the N549 of hRIG-I. Next, we purified GST-hRIG-I, GST-hRIG-I-D495, PPAT, and its deamidase-deficient mutant (PPAT-ED), and performed *in vitro* deamidation. Given that hRIG-I-D495 was deamidated at N549 when expressed and purified from 293T stable cell line, we transiently expressed hRIG-I-D495 in 293T cells, aiming to exhaust endogenous PPAT such that the majority of hRIG-I-D495 is not deamidated at N549. As analyzed by two-dimensional gel electrophoresis, transiently expressed hRIG-I-D495 migrated to the negative pole of the gel strip compared with hRIG-I-DD (Fig. S4D), indicating that the majority of hRIG-I-D495 was not deamidated at N549 in transiently transfected 293T cells. Two-dimensional gel electrophoresis of *in vitro* deamidation reaction showed that PPAT, but not PPAT-ED, shifted hRIG-I-D495 toward the positive pole of the gel strip (Fig. 5E). In contrast, hRIG-I was not shifted by PPAT or PPAD-ED. These results collectively support the conclusion that PPAT deamidates hRIG-I-D495, supporting the sequential deamidation of hRIG-I by viral UL37 and cellular PPAT.

### Deamidation by PPAT is required for evasion of RIG-I during HSV-1 infection.

**FIG 6:**
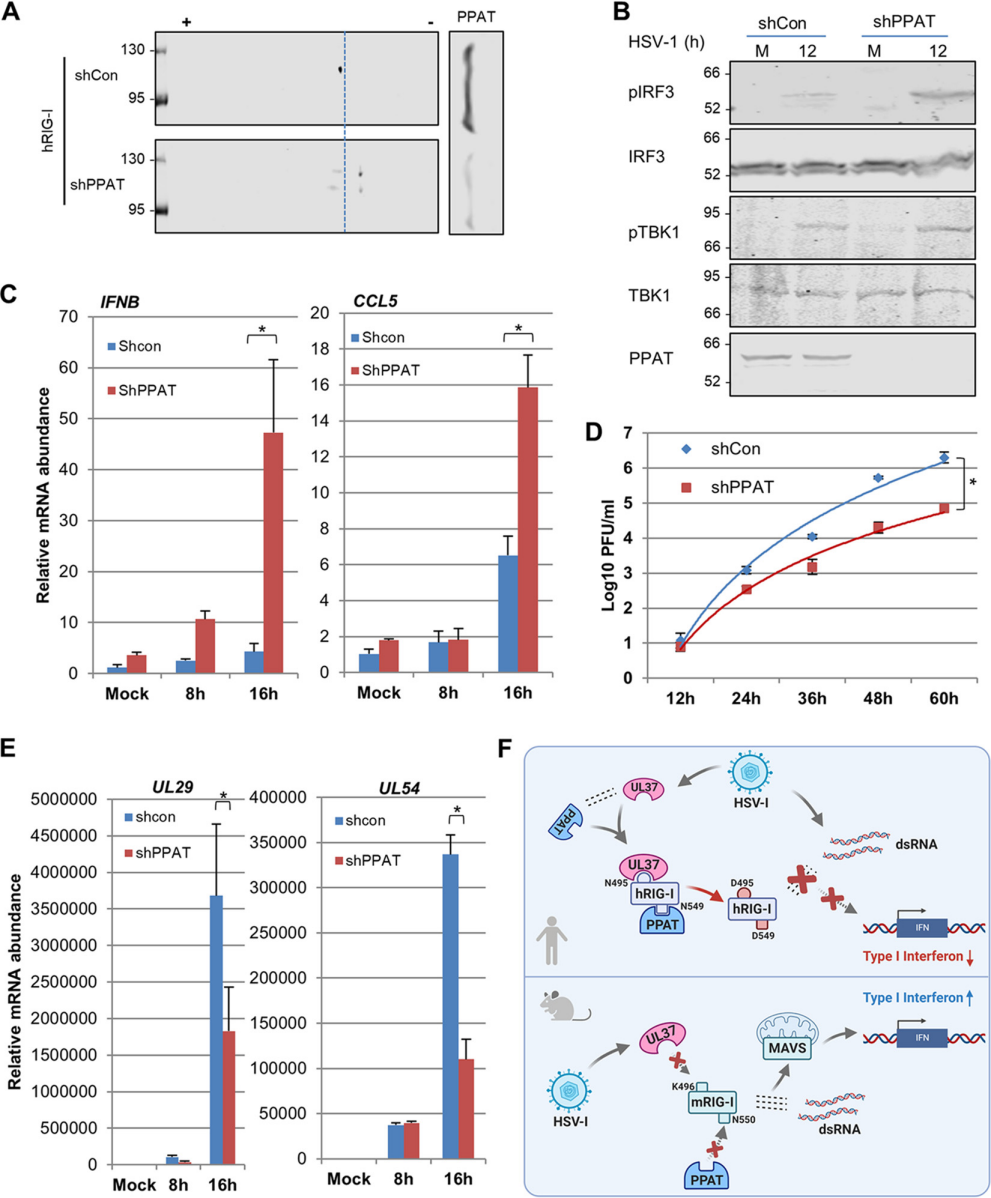
PPAT is crucial for HSV-1 to evade RIG-I-dependent innate immune response. (A) 293T cells stably expressing FLAG-hRIG-I were infected with lentivirus containing control shRNA (shCon) or PPAT shRNA (shPPAT) for 48 h, and with HSV-1 (MOI = 2) for 12 h. Whole-cell lysates (WCLs) were analyzed by two-dimensional gel electrophoresis (RIG-I, left) or regular SDS-PAGE (PPAT, right) and immunoblotting. (B and C) 293T cells were infected with lentivirus containing shCon or shPPAT, and with HSV-1 as described in panel A. WCLs were prepared at 12 h postinfection and analyzed by immunoblotting with the indicated antibodies (B). (C) Total RNA was extracted at the indicated time points, and cDNA was generated and analyzed by real-time PCR with primers specific for *IFNB* and *CCL5* (C). (D and E) Viral titer (D), HSV-1 *UL29* and *UL54* abundance (E) in cells as described in panel A and infected with HSV-1 (MOI = 0.01 and 2, respectively) for the indicated time. (F) A schematic model illustrating the species-specific deamidation of RIG-I and the collaborative action between viral UL37 deamidase and the cellular PPAT deamidase in HSV-1 lytic replication. Data are presented as mean ± SD. Significance was calculated using unpaired (paired for Fig. 6D), two-tailed Student's *t* test. *, *P < *0.05; **, *P < *0.01; ***, *P < *0.001; NS, nonsignificant.

Our findings collectively support the conclusion that HSV-1 UL37 and cellular PPAT target N495 and N549, respectively, for deamidation. To determine the biological significance of PPAT in RIG-I deamidation and innate immune evasion, we examined hRIG-I charge change and innate immune signaling. Indeed, depletion of PPAT shifted hRIG-I toward the negative pole of the gel strip in HSV-1-infected cells, suggesting that PPAT depletion impedes hRIG-I deamidation induced by HSV-1 (Fig. 6A). Next, we determined the effect of PPAT depletion on RIG-I-mediated innate immune activation during HSV-1 infection. Indeed, transient knockdown of PPAT elevated the phosphorylation of TBK-1 and IRF3 (Fig. 6B). This also correlated with increased expression of antiviral genes, such as *IFNB* and *CCL5* (Fig. 6C). Importantly, when PPAT was depleted, cells were supplemented with hypoxanthine, a metabolite that can be directly converted into IMP, the end product of the *de novo* purine synthesis pathway. As such, transient depletion of PPAT had no significant effect on 293T cells as cell viability was assessed (data not shown). Mass spectrometry analysis indicates that PPAT depletion did not significantly alter the intracellular concentrations of the majority of metabolites of the central carbon metabolism and nucleotide synthesis (see Fig. S5A in the supplemental material). In fact, it increased the intracellular concentration of adenine and xanthosine, while the levels of IMP, AMP, and GMP were not significantly reduced (Fig. S5B). Consistent with elevated antiviral immune response against HSV-1, PPAT depletion reduced HSV-1 lytic replication (Fig. 6D), which also correlated with lower levels of viral lytic transcripts as analyzed by real-time PCR (Fig. 6E). These results collectively support the conclusion that loss of PPAT impairs the evasion of RIG-I-dependent innate immune activation during HSV-1 infection.

10.1128/mBio.00115-21.5 FIG S5 Related to Fig. 6. (A) The metabolic pathway map depicting the log_2_ fold change (FC) (shPPAT/shControl) of intracellular pool sizes of metabolites in glycolysis, tricarboxylic acid (TCA) cycle, pentose phosphate pathway, and nucleotide synthesis using the indicated color scale. Metabolites that were not measured are shown as small circles with black. The sizes of circle correspond to their transformed *P* values in the metabolic pathway. (B) Relative IMP, AMP, and GMP from the WCLs of 293T/RIG-I stable cells transfected with control or shRNA targeting PPAT for 72 h and analyzed by tandem mass spectrometry. Data are presented as mean ± SD. Significance was calculated using a two-tailed, unpaired Student’s *t* test. NS, nonsignificant. Download FIG S5, PDF file, 0.2 MB. Copyright © 2021 Huang et al. 2021 Huang et al. https://creativecommons.org/licenses/by/4.0/ This content is distributed under the terms of the Creative Commons Attribution 4.0 International license.

## DISCUSSION

Signaling cascades underpinning innate immune defense are ubiquitous and highly conserved. However, species-specific virus-host interactions that regulate or manipulate innate immune signaling, particularly those entailing highly conserved signaling molecules such as RIG-I and cGAS, are not well studied. We explore herpesvirus immune evasion to examine the mechanism of regulation specific to mammalian species. We report here that HSV-1 targets an asparagine residue, N495, of hRIG-I for deamidation. Remarkably, mRIG-I contains a lysine residue at the equivalent position to that in RIG-I proteins of human and nonhuman primates, thus rendering mRIG-I with resistance to deamidation induced by HSV-1. As such, mRIG-I is more potent than hRIG-I in restricting HSV-1 lytic replication. Although it is not surprising that mice deficient in RIG-I are more susceptible to HSV-1 infection than wild-type mice, this study for the first time demonstrates *in vivo* antiviral activity of RIG-I against a herpesvirus that carry a DNA genome. These results are consistent with the findings that RIG-I detects RNA molecules that are derived from cellular and viral DNAs during infection, to induce innate immune cytokine production (16, 17, 37). The observations that the hRIG-I-K495 mutant and mRIG-I are resistant to UL37-mediated deamidation, activate innate immune signaling events, and restrict HSV-1 replication more robustly than the hRIG-I wild type lend unequivocal credence to the antiviral role of RIG-I and viral evasion thereof in HSV-1 infection.

Cellular GATs are known for their metabolic functions in cell proliferation and homeostasis (33). The findings that these cellular metabolic enzymes can deamidate signaling molecules such as RIG-I (41) and RelA (35) support the hypothesis that cellular GATs may couple key signaling pathways to the metabolic status of a cell. Further supporting this hypothesis, deamidated RelA appears to facilitate aerobic glycolysis via inducing the expression of checkpoint enzymes of this pathway, while shutting down inflammatory gene expression (35). In this study, we found that PPAT, the rate-limiting enzyme of the *de novo* purine synthesis pathway, can deamidate a residue within the helicase domain of RIG-I. Consequently, deamidated RIG-I failed to bind dsRNA and to hydrolyze ATP in the presence of dsRNA, a process that likely promotes the oligomerization and signaling activation downstream of RIG-I (38). Thus, this finding adds PPAT to the growing list of cellular deamidases that are genuine metabolic enzymes of the GAT family and describes new functions of PPAT in innate immune regulation. It is very likely that other cellular and viral proteins are deamidated by PPAT. Future investigation will identify and characterize these deamidation events in regulating various signaling pathways, such as the innate immune response. Given the rate-limiting role of PPAT in *de novo* purine synthesis, these studies will reveal mechanisms of key metabolic enzymes in coupling purine synthesis with other fundamental biological processes. It is of note that RIG-I appears to interact with another GAT, CTPS1. However, knockdown of CTPS1 failed to impede deamidation of RIG-I, implying a potential deamidase-independent regulation of RIG-I by CTPS1 or *vice versa*. It will be interesting to investigate the biological significance of this interaction.

In resting cells, hRIG-I is minimally deamidated at N495 and N549 in the absence of HSV-1 infection. Interestingly, mRIG-I is resistant to HSV-1-induced deamidation, although mRIG-I shares N549 with hRIG-I. Comparative and mutational analyses identify N495 that is sufficient and necessary to confer hRIG-I with the sensitivity of deamidation to HSV-1 UL37. This work supports a two-step deamidation model, in which HSV-1 UL37 targets N495 of hRIG-I for deamidation (16), which is followed by PPAT-mediated deamidation of N549 (Fig. 6F). Such a model is substantiated by the following observations. First, hRIG-I-D495, but not wild-type hRIG-I, is deamidated at N549 in the absence of HSV-1 infection, as analyzed by two-dimensional gel electrophoresis and mass spectrometry, supporting the deamidase activity of cellular origin. Indeed, hRIG-I-D495 interacts with PPAT, and knockdown of PPAT impedes the deamidation of N549 of hRIG-I-D495. Second, knockdown of PPAT also impedes hRIG-I deamidation in HSV-1-infected cells, supporting the distinct contribution of HSV-1 UL37 and cellular PPAT in hRIG-I deamidation. This result also implicates the crucial role of N549 deamidation in preventing RIG-I in sensing RNA and activating innate immune signaling events. Third, UL37 expression shifted hRIG-I in cells much further than it did in test tube, which agrees with the initial deamidation of N495 by UL37 and subsequent deamidation by cellular PPAT in cells. As analyzed by *in vitro* deamidation assay, UL37 only deamidates N495 of hRIG-I. Finally, *in vitro* deamidation assay unequivocally shows that hRIG-I-D495, but not wild type hRIG-I, is deamidated by PPAT, indicating that PPAT is a *bona fide* deamidase targeting N549. Thus, UL37 and PPAT collaborate to deamidate hRIG-I in muting its RNA-sensing activity in HSV-1-infected cells. The sequential and coordinated actions of these deamidases are reminiscent of the priming mechanism in regulating NFAT activation by phosphorylation (39, 40). Whether there is a cellular deamidase that targets N495 of hRIG-I for deamidation remains unknown. The identification of such a deamidase will certainly reveal important regulatory roles of deamidation in cellular and molecular biology.

## MATERIALS AND METHODS

### Two-dimensional gel electrophoresis.

Cells (1 × 10^6^) were lysed via resuspension in 150 μl rehydration buffer (8 M urea, 2% CHAPS (3-[(3-cholamidopropyl)-dimethylammonio]-1-propanesulfonate), 0.5% IPG buffer, and 0.002% bromophenol blue) and three pulses of sonication. Cell lysates were centrifuged at 20,000 × *g* and 4°C for 15 min. Centrifuged cell lysates were loaded to isoelectric focusing (IEF) strips for first-dimension electrophoresis with a program comprising the following: 20 V, 10 h (rehydration); 100 V, 1 h; 500 V, 1 h; 1,000 V, 1 h; 2,000 V, 1 h; 4,000 V, 1 h; and 8,000 V, 4h. After IEF, strips were incubated in SDS equilibration buffer (50 mM Tris-HCl [pH 8.8], 6 M urea, 30% glycerol, 2% SDS, and 0.001% bromophenol blue) containing 10 mg/ml 1,4-dithiothreitol (DTT) for 15 min and SDS equilibration buffer containing 2-iodoacetamide for 15 min. Strips were washed with SDS-PAGE buffer, resolved by SDS-PAGE, and analyzed by immunoblotting.

### *In vitro* deamidation assay.

GST-hRIG-I, GST-hRIG-I-D495, PPAT, and the PPAT-ED mutant were purified from transfected 293T cells to homogeneity as determined by silver staining. *In vitro* on-column deamidation of hRIG-I was performed as previously reported (16, 27, 41). Briefly, ∼0.2 μg of PPAT or PPAT-ED and 0.6 μg of GST-hRIG-I or GST-hRIG-I-D495 (bound to glutathione-conjugated agarose) were added to a total volume of 30 μl. The reaction was carried out at 37°C for 45 min in deamidation buffer (50 mM Tris–HCl at pH 7.2, 1 mM KCl, 5 mM phosphoribosyl 5′-pyrophosphate, and 15 mM MgCl_2_). Protein-bound glutathione *S*-transferase (GST) beads were washed with deamidation buffer, and GST-hRIG-I or GST-hRIG-I-D495 was eluted with rehydration buffer (8 M urea, 2% CHAPS, 0.5% IPG buffer, an 0.002% bromophenol blue) at room temperature. Samples were then analyzed by two-dimensional gel electrophoresis and immunoblotting.

### Cell lines and viruses.

HEK293T, Vero, and mouse embryonic fibroblasts (MEFs) were cultured in Dulbecco’s modified Eagle’s medium (DMEM; Corning) supplemented with 10% heat-inactivated fetal bovine serum (FBS; HyClone), penicillin (100 U/ml), and streptomycin (100 μg/ml). Wild-type and *Rig-i^−/−^* MEFs were prepared as described previously (42). Wild-type HSV-1 (KOS strain) and recombinant HSV-1 were generated and amplified in Vero cells as previously described (16), with viral titers ranging from 10^7^ to 10^8^ PFU/ml. Sendai virus was purchased from Charles River Laboratories.

### Constructs.

Mammalian expression plasmids for RIG-I and its mutants were described previously (7, 43–45). Control shRNA and shRNA specific for human RIG-I and glutamine amidotransferases were purchased from Thermo Scientific. Mammalian transient expression plasmids and lentiviral stable expression plasmids for RIG-I, UL37, and glutamine amidotransferase were generated by standard molecular biology techniques. All point mutants, including those of RIG-I, UL37, and glutamine amidotransferases, were generated by site-directed mutagenesis and confirmed by sequencing.

### Antibodies and reagents.

Antibody against UL37 was generated by Cocalico Biologicals, Inc. Antibodies against IRF3 (FL-425), GST (Z-5), and RIG-I (H-300) were purchased from Santa Cruz Biotechnology. Antibodies against FLAG (M2; Sigma), V5 (A190-220A; Bethyl Group), CAD (A1301-374A; Bethyl Group), PPAT (15401-1-AP; Proteintech), CTPS1 (15914-1-AP; Proteintech), MAVS (5-20337; Thermo Scientific), RIG-I (SS1A; Enzo Life Sciences), p-S172 TBK-1 (D52C2; Cell Signaling), TBK-1 (3013S; Cell Signaling), p-S396 IRF3 (4D4G; Cell Signaling), and β-actin (Ab8226; Abcam) were purchased from the indicated suppliers.

### DNA transfection.

For plasmid transfection using HEK293T cells, the calcium phosphate transfection method was applied. Briefly, cells were seeded overnight to reach a confluence of ∼50% to 60%, and fresh medium was changed prior to the transfection. Solution containing calcium chloride mixed with plasmids (10 to 15 μg for a 10-cm plate) was dropwise added to HEPES-buffered saline containing sodium phosphate upon vortexing. After appropriate incubation, the DNA-calcium phosphate precipitate was then added to cells. Medium was replaced at 6 h posttransfection, and cells were incubated for 24 to 48 h before harvest.

### Lentivirus-mediated stable cell line construction.

Lentiviruses were produced as previously described (44, 46). Briefly, HEK293T cells were transfected with two packaging plasmids, VSV-G and DR8.9, and pCDH lentiviral expression vector or lentiviral shRNA plasmids. At 48 h posttransfection, lentivirus-containing medium was filtered and concentrated by centrifugation if necessary. HEK293T cells and MEFs were infected with lentivirus-containing medium in the presence of Polybrene (8 μg/ml) with centrifugation at 1,800 rpm for 45 min. Cells were selected at 48 h postinfection and maintained in 10% FBS-DMEM supplemented with puromycin (∼1 to 2 μg/ml).

### Plaque assay.

HSV-1 and vesicular stomatitis virus (VSV) titers were determined by plaque assay on Vero monolayer as previously described (47). Briefly, 10-fold serially diluted virus-containing medium was added to Vero cells and incubated for 2 h at 37°C. After removing virus-containing medium, DMEM containing 2% FBS and 1% methylcellulose (Sigma) was added, and plates were placed in an incubator to allow plaque formation. Plaques were counted at 1 or 3 days postinfection for VSV and HSV-1, respectively.

### Mouse studies.

*Rig-i*^−/−^ mice and their wild-type littermate controls, with a 129 Sv × C57BL/6 genetic background, were generously provided by S. Akira (Osaka University) (48). All mice were genotyped and bred under pathogen-free conditions in the animal facility at the University of Southern California. Experiments were performed with approval from the University of Southern California Institutional Animal Care and Use Committee. Age-matched (10- to 16-week-old) and sex-matched mice were used for all experiments.

For corneal infection, age- and gender-matched *Rig-i*^−/−^ mice and their wild-type littermate controls were subjected to anesthesia with isoflurane. Upon sedation, the cornea was scratched with a needle (26 G × 3/8 in.) 10 times, and 3,200 PFU of HSV-1 in 4 μl saline was added dropwise. Mouse eyes were then closed and massaged to facilitate viral absorption. Mice were again subjected to anesthesia with isoflurane at the indicated days postinfection, when eyes were swabbed with cotton swabs for 10 times. The cotton swabs were then stored in DMEM, and HSV-1 viral particles were released by vortexing. Viral titer was measured by plaque assay as described above.

For survival analysis, age- and gender-matched *Rig-i*^−/−^ mice and their wild-type littermate controls were infected with 1 × 10^6^ PFU of HSV-1 via intraperitoneal injection. Mouse survival was monitored daily for 10 days. Mice were weighed and observed for clinical signs of disease daily and were humanely euthanized if they lost more than 25% of their starting weight or exhibited severe clinical signs of disease, including paralysis, lethargy, and hunched posture.

### Protein expression and purification.

HEK293T cells were transfected with expression vector containing FLAG-tagged gene of interest. At 48 h posttransfection, cells were harvested and lysed with Triton X-100 buffer (20 mM Tris [pH 7.5], 150 mM NaCl, 1.5 mM MgCl_2_, 20 mM β-glycerophosphate, 1 mM sodium orthovanadate, 10% glycerol, 0.5 mM EGTA, and 0.5% Triton X-100) supplemented with a protease inhibitor cocktail (Roche). Whole-cell lysates were sonicated and centrifuged at 12,000 rpm and 4°C for 15 min. The supernatant was collected, filtered, precleared with Sepharose 4B beads at 4°C for 1 h, and then incubated with anti-FLAG agarose beads at 4°C for 4 h. The agarose beads were washed extensively and eluted with 0.2 mg/ml 3×FLAG peptide. The eluted proteins were analyzed by SDS-PAGE and immunoblotting.

### Coimmunoprecipitation and immunoblotting.

For coimmunoprecipitation (Co-IP) using exogenous protein, HEK293T cells were transfected with indicated expression plasmids for 48 h. For Co-IP using endogenous proteins, cells were directly harvested. Whole-cell lysates (WCLs) were prepared with NP-40 buffer (50 mM Tris-HCl [pH 7.4], 150 mM NaCl, 1% NP-40, 5 and mM EDTA) supplemented with 20 mM β-glycerophosphate and 1 mM sodium orthovanadate. WCLs were then sonicated, centrifuged, and precleared with Sepharose 4B for 1 h. Precleared samples were incubated with indicated antibodies overnight and with protein A/G agarose for 1 h at 4°C, or with antibody/glutathione-conjugated agarose for 4 h at 4°C. Agarose beads were washed extensively, and samples were eluted with SDS-PAGE loading buffer at 95°C for 10 min. The precipitated proteins were analyzed by SDS-PAGE and immunoblotting.

Immunoblotting was performed with the corresponding primary antibodies (1:1,000 dilution), as indicated in each figure, and IRDye800-conjugated secondary antibodies (1:10,000 dilution; LI-COR), unless specified otherwise. Proteins were visualized with an Odyssey infrared imaging system (LI-COR).

### Quantitative real-time PCR.

Quantitative real-time PCR (qRT-PCR) was performed as previously described (41). Cells were infected with viruses or treated with agents for indicated time period. Total RNA was extracted using TRIzol reagent (Invitrogen). Complementary cDNA was synthesized from DNase I-treated total RNA using reverse transcriptase (Invitrogen). Complementary cDNA was diluted and qRT-PCR was performed using SYBR green mastermix (Applied Biosystems) by real-time PCR instrument (Applied Biosystems). Relative mRNA expression for each target gene was calculated by the threshold cycle (2^−ΔΔ^*^CT^*) method, using β-actin as an internal control. Sequences of qRT-PCR primers are listed in Table 1.

**TABLE 1:** qRT-PCR primers used in this study

Primer target	Primer	Sequence
Human β-actin	Forward	5′-CTGGCACCCAGCACAATG-3′
	Reverse	5′-GCCGATCCACACGGAGTACT-3′
Human IFN-β	Forward	5′-AGGACAGGATGAACTTTGAC-3′
	Reverse	5′-TGATAGACATTAGCCAGGAG-3′
Human ISG56	Forward	5′-TCTCAGAGGAGCCTGGCTAA-3′
	Reverse	5′-TGACATCTCAATTGCTCCAG-3′
Human CCL5	Forward	5′-CCTGCTGCTTTGCCTACATTGC-3′
	Reverse	5′-ACACACTTGGCGGTTCTTTCGG-3′
Mouse β-actin	Forward	5′-ACGGCCAGGTCATCACTATTG-3′
	Reverse	5′-CAAGAAGGAAGGCTGGAAAAGA-3′
Mouse IFN-β	Forward	5′-TCCGAGCAGAGATCTTCAGGAA-3′
	Reverse	5′-TGCAACCACCACTCATTCTGAG-3′
Mouse ISG56	Forward	5′-ACCATGGGAGAGAATGCTGAT-3′
	Reverse	5′-GCCAGGAGGTTGTGC-3′
Mouse CXCL10	Forward	5′-CTCATCCTGCTGGGTCTGAG-3′
	Reverse	5′-CCTATGGCCCTCATTCTCAC-3′
HSV-1 UL29	Forward	5′-AAGCTGGTTGCGTTGGAG-3′
	Reverse	5′-TTTCTGCTGAAGCAGTTCCA-3′
HSV-1 UL54	Forward	5′-GTCCTGCGCTCCATCTCC-3′
	Reverse	5′-GTCGTGCATGACCTGTGC-3′

### Mass spectrometry analysis.

For identification of deamidation sites, FLAG-RIG-I, FLAG-RIG-I-D495, and FLAG-RIG-I-D549 were purified with anti-FLAG-conjugated agarose beads for 4 h at 4°C. Beads were then extensively washed, and RIG-I, RIG-I-D495, and RIG-I-D549 were eluted with SDS-PAGE loading buffer at 95°C for 10 min. Purified RIG-I, RIG-I-D495, and RIG-I-D549 were subjected to SDS-PAGE electrophoresis and Coomassie staining. Protein bands were excised for in-gel digestion and mass spectrometry analysis.

### Metabolite extraction and analysis by liquid chromatography-mass spectrometry.

For extraction of intracellular metabolites, cells were washed with 1 ml ice-cold 150 mM ammonium acetate (NH_4_AcO [pH 7.3]) followed by addition of 1 ml of prechilled 80% methanol. Cells were incubated at −80°C for 20 min, then scraped off and transferred to new microcentrifuge tubes. The resulting methanol mixture was vortexed and centrifuged at 15,000 × *g* for 10 min at 4°C to pellet debris. The supernatant was transferred to new microcentrifuge tubes and dried with a vacuum concentrator. Metabolites were resuspended in liquid chromatography-mass spectrometry (LC-MS) grade water and subjected to LC-MS analysis. The Q-ExactivePlus Orbitrap coupled to a Vanquish Horizon ultra-high-performance LC system was used for metabolites analysis (analytical instrument supplied by Thermo Fisher Scientific). Samples were randomized and analyzed using a hydrophilic interaction LC (HILIC) approach. The LC column used was the Luna 3-μm NH2 100-Å (150- × 2.0- mm) column (Phenomenex). Mobile phase A was 5 mM NH4AcO (pH 9.9) and mobile phase B was acetonitrile. The following linear gradient with a flow rate of 0.3 ml/minute was used: 15% A for 7 min, followed by an increase to 95% A in 18 min, with a further isocratic step for 9 min, after which the column was reequilibrated for 7 min. The mass spectrometer was run in polarity switching mode (+3.0 kV/−2.5 kV) using a Fourier transform MS (FTMS) full scan of 65 to 975 *m/z* with a resolution of 70,000 ppm. Data were acquired and quantified based on the retention time and accurate mass (≤5 ppm) using TraceFinder 4.1 (Thermo Fisher Scientific) against known external standards. Metabolite levels were further normalized to the cell number in each group and compared using a two-tailed, unpaired Student’s *t* test. All samples were run in biological replications (*n* = 4). Metabolic pathway maps were made with Cytoscape software (49).

### Statistical analysis.

Statistical analysis was performed by unpaired, two-tailed Student’s *t* test. A *P* value of less than 0.05 is considered statistically significant. *, *P < *0.05; **, *P < *0.01; ***, *P < *0.001.
