# Supplementary figures and images for: Species-Specific Deamidation of RIG-I Reveals Collaborative Action between Viral and Cellular Deamidases in HSV-1 Lytic Replication
Source: mBio. 2021 Mar 30;12(2):e00115-21. doi: 10.1128/mBio.00115-21 (PMC8092204; doi:10.1128/mBio.00115-21)

Figure S1, related to Figure 1

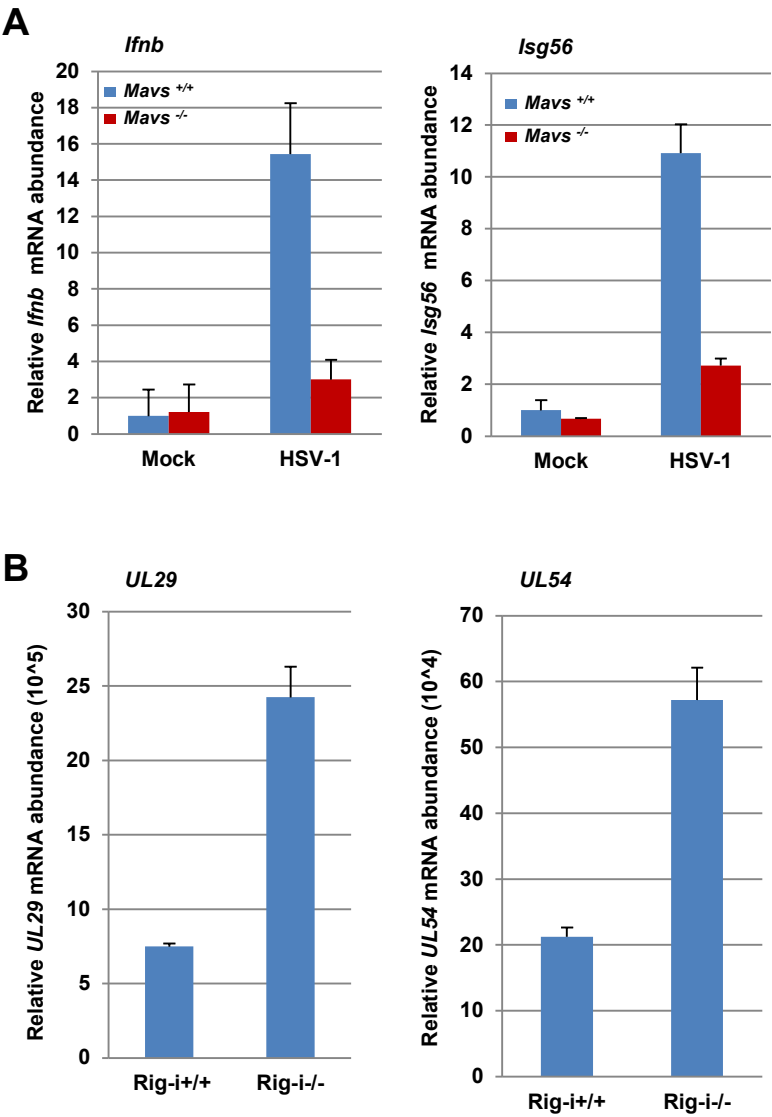

Supplement: FIG S1 [file mBio.00115-21-sf001.pdf]

Figure S2, related to Figure 3

A

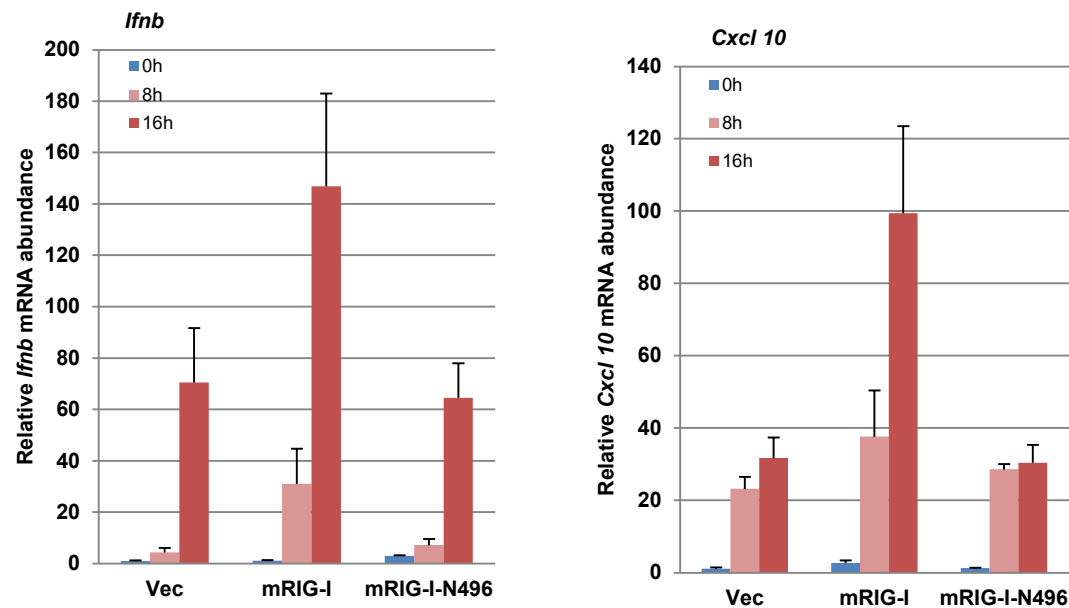

B

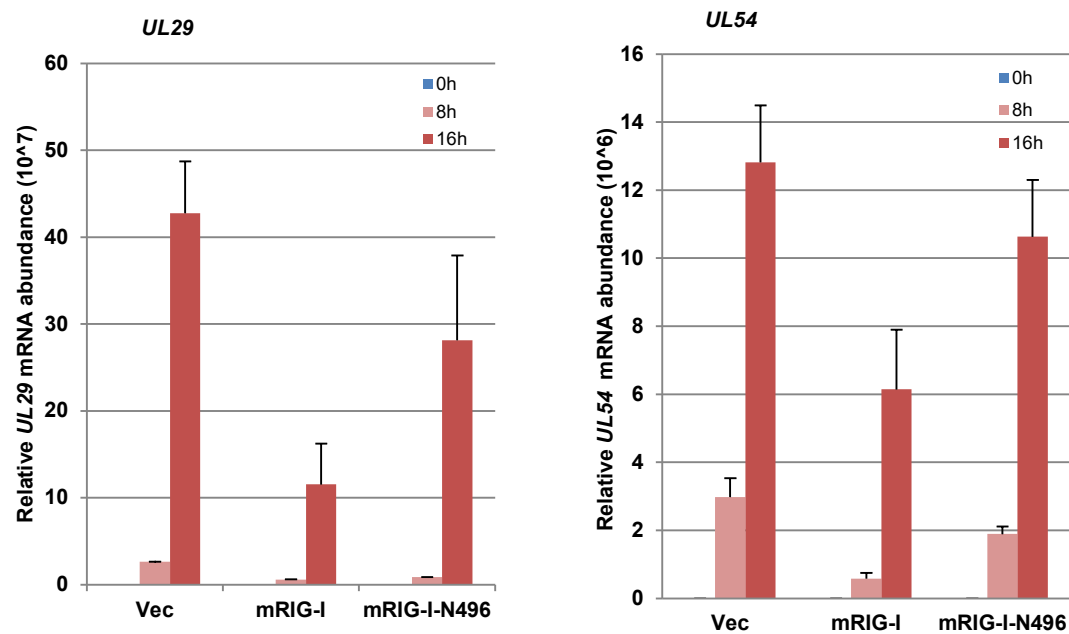

Supplement: FIG S2 [file mBio.00115-21-sf002.pdf]

Figure S3, related to Figure 4

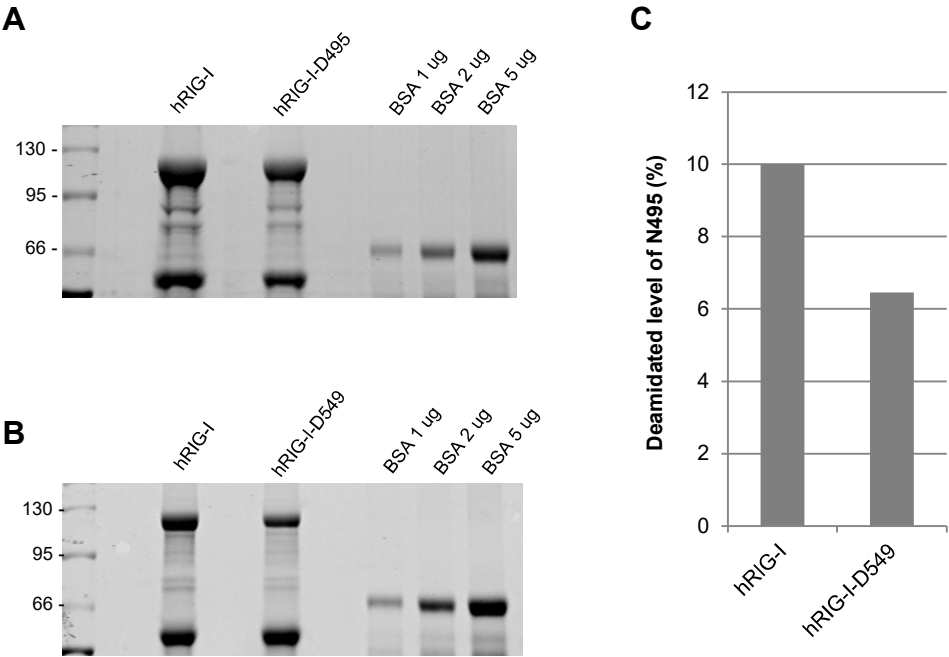

Supplement: FIG S3 [file mBio.00115-21-sf003.pdf]

Figure S4, related to Figure 5

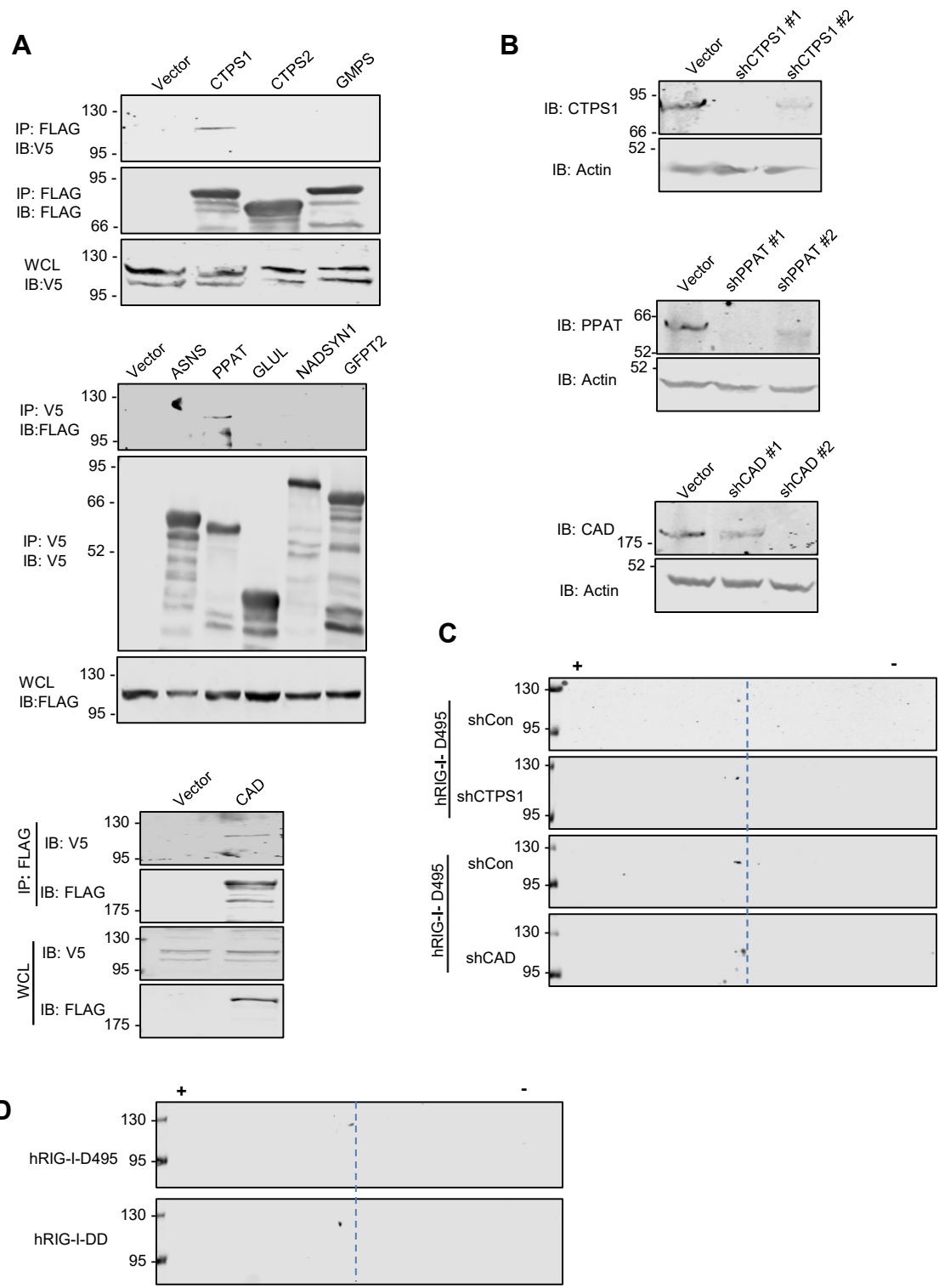

Supplement: FIG S4 [file mBio.00115-21-sf004.pdf]

Figure S5, related to Figure 6

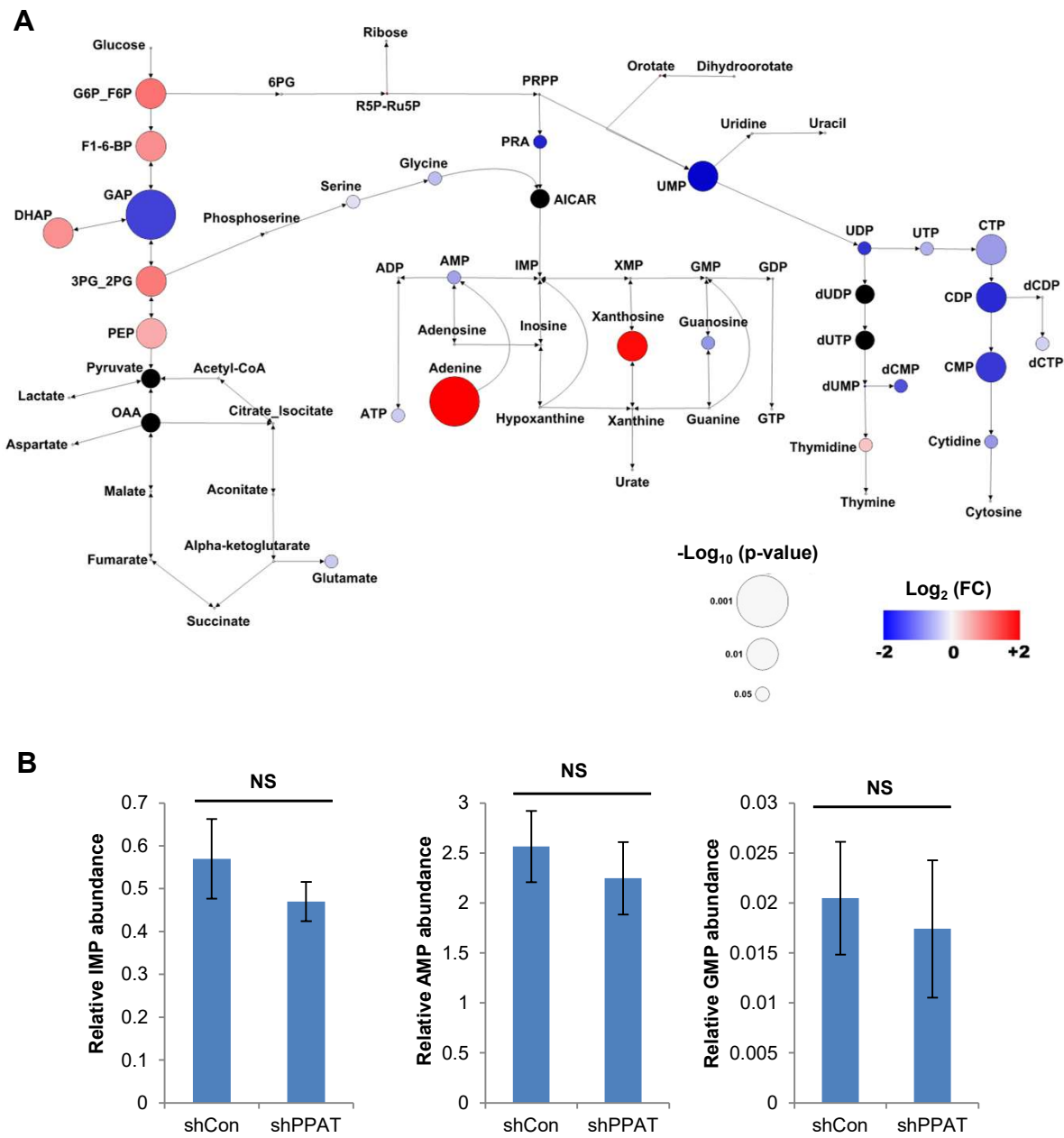

Supplement: FIG S5 [file mBio.00115-21-sf005.pdf]
